# Cardiovascular risk communication strategies in primary prevention. A systematic review with narrative synthesis

**DOI:** 10.1111/jan.15327

**Published:** 2022-06-19

**Authors:** Stacey D. Schulberg, Amy V. Ferry, Kai Jin, Lucy Marshall, Lis Neubeck, Fiona E. Strachan, Nicholas L. Mills

**Affiliations:** ^1^ BHF Centre for Cardiovascular Science The University of Edinburgh Edinburgh UK; ^2^ Usher Institute The University of Edinburgh Edinburgh UK; ^3^ Critical Care Research Group NHS Lothian Edinburgh UK; ^4^ School of Health and Social Care Edinburgh Napier University Edinburgh UK

**Keywords:** cardiovascular diseases, communication, heart disease risk factors, literature review, nursing, primary prevention, systematic review

## Abstract

**Aim:**

To evaluate the effectiveness of cardiovascular risk communication strategies to improve understanding and promote risk factor modification.

**Design:**

Systematic review with narrative synthesis.

**Data sources:**

A comprehensive database search for quantitative and qualitative studies was conducted in five databases, Cumulative Index to Nursing and Allied health Literature (CINAHL), Medical Literature Analysis and Retrieval System Online (MEDLINE), EMBASE, Applied Social Sciences Index and Abstracts (ASSIA) and Web of Science. The searches were conducted between 1980 and July 2019.

**Review methods:**

The systematic review was conducted in accordance with Cochrane review methods. Data were extracted and a narrative synthesis of quantitative and qualitative results was undertaken.

**Results:**

The abstracts of 16,613 articles were assessed and 210 underwent in‐depth review, with 31 fulfilling the inclusion criteria. We observed significant heterogeneity across study designs and outcomes. Nine communication strategies were identified including numerical formats, graphical formats, qualitative information, infographics, avatars, game interactions, timeframes, genetic risk scores and cardiovascular imaging. Strategies that used cardiovascular imaging had the biggest impact on health behaviour change and risk factor modification. Improvements were seen in diet, exercise, smoking, risk scores, cholesterol and intentions to take preventive medication.

**Conclusion:**

A wide range of cardiovascular risk communication strategies has been evaluated, with those that employ personalized and visual evidence of current cardiovascular health status more likely to promote action to reduce risk.

**Impact:**

Future risk communication strategies should incorporate methods to provide individuals with evidence of their current cardiovascular health status.

## 1 INTRODUCTION

Cardiovascular disease is the leading cause of death and disability globally (World Health Organisation, 2017). An ageing population and increases in cardiovascular risk factors, such as obesity, are exacerbating the problem (Timmis et al., 2018). It is estimated that as many as 80% of these deaths are preventable (World Health Organisation, 2017), highlighting the importance of prevention in reducing the number of unnecessary deaths and the burden of cardiovascular disease. Nurses form the largest health professional group managing cardiovascular risk factors (Hayman et al., 2015) and thus have a key role to play in the prevention of cardiovascular disease. Identifying individuals who would benefit from risk factor management is challenging due to the insidious nature of atherosclerosis, which is often advanced before symptoms develop (Cooney et al., 2009). Cardiovascular risk prediction assessments aid health professionals in clinical decision‐making about preventative treatment and are also used to inform individuals about their risks (Rossello et al., 2019). An abundance of research exists on how to accurately predict cardiovascular risk, but attention is needed on how best to inform individuals of that risk (Ahmed et al., 2012). Individuals can only make informed decisions around risk reduction if they fully comprehend their risk.

### 1.1 Background

Risk communication is the open, real‐time exchange of information, advice and opinion between experts and those at risk to improve understanding and facilitate informed decisions about clinical management (Thomson et al., 2015). It is a cornerstone of cardiovascular screening and should enhance a person’s knowledge and perception of risk, allowing them to make informed decisions (Ahmed et al., 2012). Individuals who are better informed about their cardiovascular health are more likely to adhere to preventative measures and may have better outcomes (Thomson et al., 2015). Information must be credible, clear and easy to understand (Ahmed et al., 2012) to avoid potential misinterpretation of risk and suboptimal choices about treatment. Furthermore, poor communication can also reduce confidence in health professionals and lead to anxiety and other adverse outcomes.

Many different strategies exist to communicate cardiovascular risk to individuals including numerical formats, qualitative information, visual representation or a combination. Recently, health professionals have been providing feedback from medical imaging to communicate risk information to individuals (Hollands et al., 2010). With such a pivotal role in managing risk factors, nurses involved need to be confident and skilled communicators. Understanding the effectiveness of available strategies will enable nurses to select the best approach for cardiovascular risk discussions.

## 2 THE REVIEW

### 2.1 Aims

The aim of this systematic review was to identify existing cardiovascular risk communication strategies and to evaluate their acceptability and effectiveness to improve understanding and promote risk factor modification in asymptomatic individuals without known cardiovascular disease.

### 2.2 Design

A systematic review of quantitative and qualitative studies was chosen to permit a more complete analysis and maximize findings. The review was conducted in accordance with the Preferred Reporting Items for Systematic Reviews and Meta‐Analysis (PRISMA) (Moher et al., 2009) and the Enhancing Transparency in Reporting the Synthesis of Qualitative Research (ENTEREQ) (Tong et al., 2012) guidelines (Appendix S2). The review protocol was registered with an international register of systematic reviews (PROSPERO ID: CRD42020204797).

### 2.3 Search methods

The systematic review search was guided by the population, intervention, comparison and study design (PICOS) criteria (Appendix S3). A comprehensive search was conducted in five online databases (CINAHL, Medline, EMBASE, ASSIA and web of science) using the following terms: risk communication, risk communication tools and risk messages, cardiovascular disease, ischaemic heart disease, atherosclerosis and risk presentation. Search terms were adapted to each database and are detailed in Appendix S1. The searches were conducted between 1980 and July 2019 and no language exclusions were applied. Relevant studies were sought from trial registries (clinical.gov) and reference lists of included studies were searched to identify additional studies. The titles and abstracts of all potentially eligible studies identified from the search were reviewed against pre‐determined inclusion and exclusion criteria by one reviewer (SS). The full texts of all potential studies were then independently screened by two reviewers (SS, AF and FS) and disagreements were resolved through discussion with a third reviewer (SS, AF and FS). Outcomes of interest were those associated with behaviour change, risk factor modification, risk knowledge and understanding, increased intentions and acceptability of the approach.

#### 2.3.1 Inclusion and exclusion criteria

Randomized trials, cohort studies and observational and qualitative studies were all eligible for inclusion. Only published research studies were sought and conference abstracts were excluded. Only primary prevention studies were eligible and studies of secondary prevention or in populations with established cardiovascular disease were excluded. Studies which communicated risks associated with cardiovascular treatments were also excluded. The target population was adults eligible for cardiovascular screening and thus studies with younger cohorts, such as college students were excluded. Studies which assessed the performance of cardiovascular risk scores and did not communicate risk to individuals were also excluded. Moreover, studies which evaluated behaviour change interventions, for example tailored smoking cessation interventions, were excluded as it cannot be ascertained if changes in outcomes were a result of the risk communication strategy or another component of the intervention.

### 2.4 Search outcome

**FIGURE 1:**
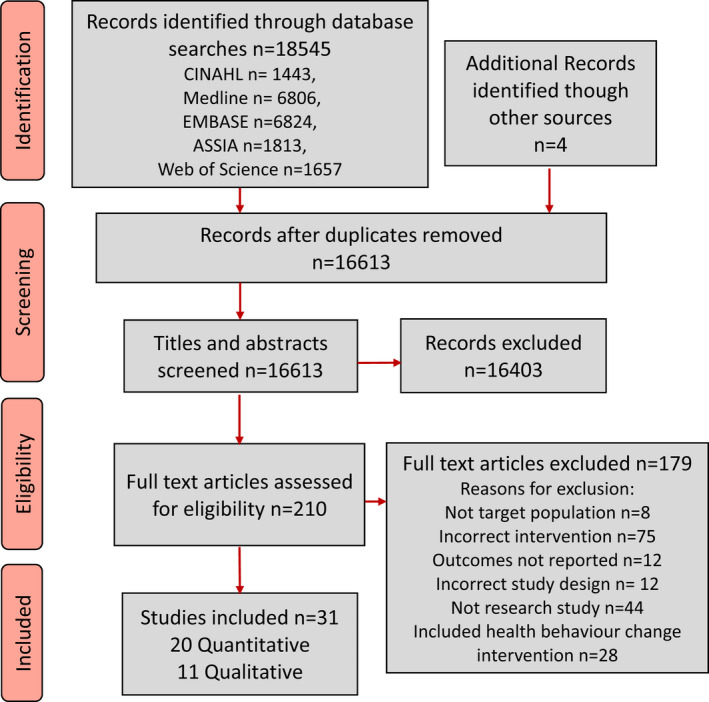
PRISMA diagram of study flow.

After duplicates were removed, a total of 16,613 titles and abstracts were identified and screened. The full‐text manuscripts for 210 studies were reviewed and a total of 31 (20 quantitative and 11 qualitative) met the inclusion criteria (Figure 1). The included studies were conducted across eight different countries from 2004 to 2019 (Table 1). A total of 20,618 (*n* = 20,256 quantitative and *n* = 362 qualitative) participants were included across the 31 studies, including patients, members of the public and general practitioners. Two studies did not report the sex of the participants, but the proportion of male participants across the remaining studies was 59%. Most of the studies provided individuals with their personal risks, but some provided hypothetical risk scenarios. Significant heterogeneity in study outcomes was observed across the quantitative studies. The outcomes assessed included acceptability of the communication strategy, emotional responses, knowledge and understanding of risk, intentions and changes in health behaviours or risk factors (Table 2). The quantitative studies evaluated the performance of the risk communication strategy against the outcomes chosen (Table 3), whereas the qualitative studies provided an exploration of the participant's perceptions of the strategy (Table 4).

**TABLE 1:** Study characteristics

Study	Author (year), Country	Study aims	Design	Sample size and context	Mean age and range	Sex (% male)	CVD risk assessment	Risk communication strategy	Variables of strategy	Professional communicating risk (or mode of delivery)	Main outcome measures
Quantitative studies	Adarkwah et al. (2019), Germany	Compare the effects of presenting a cardiovascular risk to patients and their subsequent adherence to intervention using 10‐year risk illustration in the decision aide software Arriba (emoticons) and newly developed time to event illustration	Prospective randomized trial	*n* = 294 patients who GPs wanted to discuss behaviour change with	58 30–80	42.1%	Personal risk. Absolute, Framingham risk equations	Graphical format	Coloured time to event bar graph and icon arrays (emoticons)	General physician	Adherence to behavioural change intervention patient agreed on
	Bonner et al. (2015), Australia	Test the effect of heart age on psychological and behavioural outcomes compared with 5‐year absolute risk is low‐ (i.e., 5‐year absolute risk of a CVD event <10%) to moderate‐risk (10%–15% absolute risk) patients	Randomized 2 × 3 factorial design	*n* = 469, non‐diabetic, not known to be high risk of CVD, no anti‐hypertensives or lipid lowering medication	54 45–64	50%	Personal risk. Absolute, Framingham risk equation	Numerical and graphical formats	Percentage, heart age and bar graph	Online	Intention to change lifestyle (improve diet, increase physical activity and stop smoking
	Damman et al. (2018), Netherlands	(1) Evaluate the effects of infographics about qualitative risk dimensions either with or without risk numbers on risk comprehension (2) Investigate what type of qualitative risk dimension (causes, timeline or consequences) can be best emphasized in infographics. (3) Test effects of heart age compared with traditional risk number on risk comprehension	Controlled experimental 2 × 2	*n* = 727, target population of cardiovascular risk calculators	Mean not reported 45–65	51.7%	Hypothetical scenarios. Absolute, Dutch calculator (modified version of SCORE)	Infographics, numerical formats and qualitative information	Infographics, heart age, percentages and text information	n/a hypothetical scenario	Information recall, cognitive‐risk appraisal, risk comprehension, affective risk appraisal and worry
	Domenech et al. (2016), Spain	Test the hypothesis that knowledge of the genetic risk score (GRS) in uncontrolled hypertensive patients would improve BP control	A randomized, single‐blind cohort study in two parallel groups	*n* = 67 patients with uncontrolled ambulatory BP (24 h‐ABPM >130/80)	54.5 (9.3) range not reported	74.3%	Personal risk. Genetic risk, based on Cardio in Code & SCORE	Genetic risk score	Verbal risk categories	Clinician	Improved blood pressure control
	Fair et al. (2008), UK	Test the hypothesis that responses to coronary heart disease (CHD) risk estimates are heightened by the use of ratio formats, peer group risk information and long time frames.	Cross‐sectional, between factors design	*n* = 1480, general population	Mean not reported 30–70	50%	Hypothetical scenarios. Absolute, Framingham	Numerical formats	Percentages, risk ratios and peer‐group comparators	n/a hypothetical scenario	Risk perception, emotional response and intention to change lifestyle
	French et al. (2004), UK	Examine the emotional and cognitive impact of personal and social comparison information about health risk	Observational factorial design	*n* = 970, 40–60 years with no history of heart disease	49 (6.7) range not reported	48%	Hypothetical Scenarios, Absolute risk (calculator/cohort not reported)	Numerical formats	Social comparisons, frequencies, percentages, bar graphs and icon arrays	n/a hypothetical scenario	Worry, reassurance, the likelihood of event, confidence in understanding, familiarity with cardiac events and comparison with others
	Frileux et al. (2004), France	Explore the impact of the preventive medical message on the intention to change behaviour.	Observational factorial design	*n* = 150 unpaid volunteers with no history of heart disease	Mean not reported 20–80	40.67%	Hypothetical scenarios. Absolute (calculator not defined)	Numerical formats and timeframes	Percentages and 5, 10, 15 and 20 year timeframe	n/a hypothetical scenarios	Intention to adopt a specific behaviour.
	Johnson et al. (2015), USA	Examine how knowledge of the CAC score affects risk perception, the likelihood of taking action and health‐promoting behaviour change in persons at high risk for cardiovascular disease	Observational pre‐and post‐design	*n* = 174 undergoing CAC scan with ≥3 risk factors	58.5 40–79	62%	Personal risk. Coronary calcium scoring (CAC)	Cardiovascular imaging	Coronary artery calcium scores and verbal risk category	Nurse over the telephone	Risk perceptions, the likelihood of action, worry, behaviour change and risk modification
	Kalia et al. (2006), USA	Evaluate whether visualization of coronary calcium would positively affect patients’ adherence rates	Observational pre‐ and post‐design	*n* = 505, asymptomatic patients on statin therapy referred for EBT risk assessment	61 (10) range not reported	82%	Personal risk. Electron Beam Tomography (TBT) coronary calcium scoring	Cardiovascular imaging	Coronary artery calcium scores, visualization and verbal risk categories	Research Team	Adherence to lipid‐lowering therapy and lifestyle modification
	Knowles et al. (2017), USA	Test whether providing a genetic risk score (GRS) for coronary artery disease (CAD) would serve as a motivator to improve adherence to risk‐reducing strategies	Pilot randomized control trial	*n* = 65 participants seeking cardiovascular risk evaluation with >6% risk of CAD over the next 10 years or >20% over the next 30 years	57.5 (10)* range not reported *at randomization	57%	Personal risk. Framingham risk score multiplied by genetic risk score (evaluated in ARIC cohort).	Genetic risk score	Percentage, individual percentiles on distribution graph and an absolute number of risk alleles.	Physician	Change in LDL cholesterol
	Korcarz et al. (2008), USA	Determine if identifying increased carotid intima‐media thickness (CIMT) or carotid plaque during office‐based ultrasound screening examinations could alter physicians' treatment plans and patients' motivation about health‐related behaviours	Observational pre‐and post‐design	*n* = 263 men ≥45 years or women ≥55 years with ≥1 CVD risk factor or women 45–54 with family history and ≥1 additional risk factor	58.1 45–70	48.7%	Personal risk. Ultrasound, images of the distal wall of each carotid. Plaque is defined as a thickening of intimal reflection on the arterial lumen.	Cardiovascular imaging	Carotid ultrasounds and verbal information about plaque or increased carotid intima‐media thickness	Not documented	Patient motivation and physician treatment plans
	Lopez‐Gonzalez et al. (2015), Spain	Test whether communicating cardiovascular diseases risk using the Heart Age risk assessment tool will be able to motivate a population to adopt healthier lifestyles and improve CVD risk profile over the use of a traditional percentage‐based tool	Randomized controlled trial	*n* = 2844 public sector workers	46.1 (7.1) range not reported	47.7%	Personal risk. Absolute Framingham REGICOR	Numerical formats	Percentage or heart age	Research team & clinical assistants	Framingham REGICOR score
	Naslund et al. (2019), Sweden	Investigate the impact of pictorial information about an individual’s atherosclerosis, as demonstrated by carotid ultrasound, in comparison with traditional risk factor‐based risk communication	Randomized controlled trial	*n* = 3175, aged 40 with first degree relative with CVD or aged 50 with ≥1 risk factor or aged 60.	Not reported	52.6%	Personal risk. Ultrasound of carotid intima‐media wall thickness. ARIC cohort reference for vascular age	Cardiovascular imaging	Vascular age and stylised picture of the ultrasound image. Plaque formation is shown as traffic light	Written information and images. Telephone follow‐up with a nurse.	Changes in Framingham risk score and SCORE risk score at 1 year
	Navar et al. (2018), USA	Determine how the ASCVD risk time horizon, outcome and presentation format influence risk perceptions and treatment preferences	Randomized survey study	*n* = 2708, from patient and Provider Assessment of Lipid Management (PALM) registry	Median 67 (interquartile range 61–76)	55%	Hypothesized risk scenario. The absolute risk, SCORE and ASCVD risk calculator	Numerical formats, graphical formats and timeframe icons	Lifetime or 10‐year risk timeframes, percentage, bar graph and icons.	n/a hypothetical scenarios	Perceived risk and willingness to take medication
	Orakzai et al. (2008), USA	Assess whether higher coronary artery calcium (CAC) scores determined by electron beam computed tomography (EBCT) are associated with beneficial lifestyle behaviours in asymptomatic individuals.	Observational pre‐and postdesign	*n* = 980, asymptomatic patients referred for EBCT risk assessment by GP	60 (8) range not reported	78%	Personal risk. Electron Beam Tomography (TBT) coronary calcium scoring	Cardiovascular imaging	Coronary artery calcium scores, visualization of scan and verbal risk categories	Physician and technologist	Aspirin initiation, diet changes and increased exercise
	Powers et al. (2011), USA	Evaluate the impact of personalized coronary heart disease and stroke risk communication on patients’ knowledge, beliefs and health behaviour.	Randomized controlled trial	*n* = 89, ≥55 years, diagnosis of hypertension	67 (8) range not reported	98%	Personal risk. The absolute risk, Framingham or risk factor education material	Numerical and graphical formats	Percentage, a vertical bar graph with comparison information	Not documented	Exercise, knowledge of risk factors, locus of control, medication adherence, risk estimate, worry, B.P., preferred means of reducing risk, decisional conflict and acceptability
	Ruiz et al. (2013), USA	Investigate whether icon arrays increase understanding, recall, perception of CVR and behavioural intent compared with numerical information.	Randomized controlled trial	*n* = 121 male veterans >20 years at intermediate/high risk but unaware of risk	61 (7.61) range not reported	100%	Personal risk. Absolute risk based on National Cholesterol Education Programme	Numerical and graphical formats	Frequencies, icon arrays (stick figures) and percentages	Online	Risk recall, risk change, confidence, risk perceptions, modification intentions and adherence, self‐efficacy, accessibility of information and attitudes
	Ruiz et al. (2016), USA	Compare the efficacy of a computer‐based aid communicating global CVR with or without animated avatars for improving patients, risk perception, emotional response and intention to make lifestyle changes and follow medical treatments to reduce CVR.	Randomized controlled trial	*n* = 41, male veterans >20 years at intermediate/high risk but unaware of risk.	64 (7) 49–77	100%	Personal risk. Absolute, National Cholesterol Education Programme	Avatars	Lip‐synching avatar with text or recorded voice and text	Online	Risk understanding, risk recall, risk perceptions, emotional reaction, intent to adhere to modification, self‐efficacy and attitudes towards computer aid.
	Witteman et al. (2014), USA	Test whether four icon array design factors (animated random dispersal of risk events, avatars to represent an individual, personalisation of avatar, that is choosing a colour and moving avatars) help convey randomness and how risk applies to an individual, thereby better aligning risk perceptions with risk estimates	Randomized controlled trial (2 x 2 factorial design)	*n* = 3630 of Internet users, aged 35–74, with no cardiovascular disease or previous stroke	53 (10) range not reported	45%	Personal risk. Absolute, Framingham	Avatars, graphical formats and numerical formats	Online calculator. Verbal labels, icons, avatars and frequencies.	Online	Risk perception
	Zikmund‐Fisher et al. (2014), USA	Assess whether varying the icon used in the icon arrays would alter people's risk perceptions, their recall of risk info, preferences about these graphics and assess if numeracy or graphical literacy influenced the results	Prospective randomized trial	*n* = 1504, internet users, with no known history of heart disease or stroke	53.8 (9.7) range not reported	45.7%	Personal risk. Absolute, D'Agostino model developed from Framingham	Graphical formats	Icon arrays—ovals, blocks, restroom icons, faces (smiley and frowns), head outlines and head and shoulder photographs	Online	Risk recall, risk perceptions and graph preferences
Qualitative Studies	Ancker et al. (2009), USA	Explore consumer preferences for different interactive graphics, basic usability and consumer interpretations of what they were seeing.	Focus groups	*n* = 16 members of the general public aged 20–65+	Not reported	18.75%	Hypothesized scenarios. Absolute, calculator based on the National Cholesterol Education Programme guidelines	Graphical formats and numerical formats	Online interactive calculator with bar graphs, icon arrays and frequencies	n/a hypothetical scenarios	N/A
	Bonner, Jansen, McKinn, et al. (2014); Bonner, Jansen, Newell, et al. (2014), Australia	Investigate patient experiences and understanding of online heart age calculators that use different verbal, numerical and graphical formats based on 5‐ and 10‐year Framingham risk equations used in clinical practice guidelines around the world	Semi‐structured interviews	*n* = 26 ≥ 1 CVD risk factor, not taking anti‐hypertensives or lipid lowering medication	54 40–70	38.46%	Personal risk. Absolute, Framingham risk equations	Numerical formats & timeframes	Online, heart age, absolute risk with 5 and 10 year timeline	Online	N/A
	Bonner, Jansen, McKinn, et al. (2014); Bonner, Jansen, Newell, et al. (2014), Australia	Explore GPs' descriptions of their communication strategies in CVD risk management, and investigate the reasons why they do or do not communicate quantitative absolute risk guidelines to patients.	Semi‐structured interviews	*n* = 25, GPs with varying levels of experience (<10–>30 years)	Mean not reported <40–>60	28.57%	N/A communication styles	N/A	Positive, scare tactic and indirect	n/a	N/A
	Damman et al. (2016), Netherlands	Identify the barriers from the perspective of consumers with low health literacy in using risk information as provided in cardiometabolic risk assessments	Cognitive interviews	*n* = 23, low health literacy/numeracy	52.6 40–66	45%	Actual, personal risk. Absolute, Dutch national cardiometabolic risk assessment	Numerical formats	Online, self‐assessment, percentage	Online	N/A
	Damman et al. (2017), Netherlands	Examine how lay people understand the result derived from an online cardiometabolic risk calculator.	Eye tracker and semi‐structured interviews	*n* = 16, target population of the prevention programme (no history of type 2 diabetes, CVD and CKD)	Mean not reported 45–60	19%	personal risk. Absolute, National Prevention programme for CVD, type 2 diabetes and CKD calculator	Numerical and graphical formats	Risk percentage, natural frequency, bar graph, categorical verbal label and comparative information	Online	N/A
	Goldman et al. (2006), USA	Explore patients’ perceptions of cholesterol and cardiovascular disease risk and their reactions to three strategies for communicating CVD risk	Focus groups	*n* = 50, aged 20–<70. Recruited from primary care	Not reported	57.9%	Hypothetical scenarios. Absolute, Framingham	Numerical and graphical formats	Icon arrays, bar graph, percentage and heart age	Research team	N/A
	Hill et al. (2010), Australia	Explore consumer and GP views and preferences about the most suitable formats for the representation and discussion of absolute risk for CVD.	Focus groups	*n* = 37 (19 consumers without CVD and 18 GPs)	Mean not reported 40–60	Not reported	Hypothetical scenarios. Absolute, Framingham	Numerical formats, qualitative information and graphical formats	Statements, icons, percentages, timeframes, risk ratios and frequencies	Research team	N/A
	Middlemass et al. (2014), UK	Explore how patients who have had a recent conventional cardiovascular risk assessment, perceive additional information from genetic testing for CHD	Interviews	*n* = 29, patients undergoing CVD risk assessment who wanted genetic test	Median—59 53.5–62	74.4%	Personal risk. Genetic risk, commercial test using panel of nine risk alleles	Genetic risk scores	Verbal risk category	Written	N/A
	Shefer et al. (2016), UK	Explore the short term response to receiving different forms of CHD risk information and lifestyle advice for risk reduction.	Interviews & focus groups (embedded in RCT)	*n* = 54 (interviews n = 41), blood donor study participants with no history of CVD	Mean not reported 40–80	59.3%	Personal risk. Genetic risk & phenotypic (absolute risk based on Framingham)	Numerical formats	Percentage, natural frequency, heart age, visual & peer comparative risk	Online	N/A
	Sheridan et al. (2009), USA	Explore how individuals respond to global coronary heart disease (CHD) risk and use it in combination with treatment information to make decisions to initiate and maintain risk‐reducing strategies	Focus groups	*n* = 29, known risk but no CVD	62.7 52–75	72%	Personal risk (mock risk if unable to calculate). Absolute risk, calculator not reported	Numerical and graphical formats	Percentage, coloured bar chart and comparative information	Research team	N/A
	Wan et al. (2008), Australia	Develop a model for a joint approach to its implementation based on an exploration of the views of patients, general practitioners (GPs) and key informants (KIs)	Focus groups and interviews	*n* = 57, (22 GPs, 26 patients and 9 Key informants)	Patients: 63.5 42–81	Not reported for all	Risk not provided. Absolute Risk, New Zealand CVAR calculator	N/a	Online (self‐assessment) and paper calculator	n/a	N/A

**TABLE 2:** Overview of outcomes and results

Strategy	Study		Outcomes					
			Acceptability	Emotional response	Risk knowledge and understanding	Intentions	Changes in health behaviours or risk factors	
Numerical format								
Percentage	Powers et al. (2011)		+		+	−	o	
Risk ratios	Fair et al. (2008)			−	+	+		
Heart age	Bonner et al. (2015)		−	−	+	−		
	Lopez‐Gonzalez et al. (2015)						+	
	Damman et al. (2018)				+	+		
Graphical format								
Icon arrays	Ruiz et al. (2013)				−	−		
	Zikmund‐Fisher et al. (2014)	Rest rooms and photos			+			
		Blocks and faces			−			
	Witteman et al. (2014)				+	−		
	French et al. (2004)			+				
Bar graphs	French et al. (2004)			+				
	Adarkwah et al. (2019)				+			
	Navar et al. (2018)				+	+		
Avatars	Witteman et al. (2014)				+	+		
	Ruiz et al. (2016)				o	+		
Qualitative information	Damman et al. (2018)				+			
Infographics	Damman et al. (2018)				−			
Timeframe								
Lifetime	Fair et al. (2008)			−				
Shorter timeframe (5–10 years)	Frileux et al. (2004)					+		
Genetic risk score	Domenech et al. (2016)						+	
	Knowles et al. (2017)				+		+	o^a^
Cardiovascular imaging								
Coronary artery calcium scoring	Johnson et al. (2015)						+	
	Orakzai et al. (2008)						+	
	Kalia et al. (2015)						+	
Ultrasound carotid	Näslund et al. (2019)						+	
	Korcarz et al. (2008)				+	+		

^a^ Positive effect on weight but negative effect on LDL cholesterol. 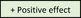; 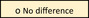; 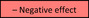.

**TABLE 3:** Quantitative results

Strategy	Study	Comparators	Results
Numerical formats			
Percentages	Powers et al. (2011)	Risk factor education	Agreed information presented more clearly than risk factor education only (57% vs. 29% *p* = 0.008*^1^). More helpful in making decisions (47% vs. 31% *p* = 0.1*^1^). Less decisional conflict over risk reduction methods (*p* = 0.003)*^2^. No differences in health behaviours, blood pressure, medication adherence or smoking. Perceived risk declined at 3 months (*p* = 0.053)*^1^. *^1^ conditional logistic regression (Fisher exact test when data sparse) *^2^ *t* test
Risk ratios	Fair et al. (2008)	Percentages	Increased risk perceptions (*p* < 0.001)*^1^. Increased intentions to make lifestyle changes (*p* = 0.047)*^2^. Increased levels of worry (*p* = 0.0004)*^2^ and disturbance (*p* = 0.001)*^2^. *^1^ logistic regression *^2^ ANOVA
Heart age	Bonner et al. (2015)	Percentages	Viewed results as less credible (*p* < 0.001*) and had less of a positive emotional response (*p* < 0.001*). No difference in intentions to change lifestyle (reduce smoking *p* = 0.67, improve diet *p* = 0.47, improve physical activity *p* = 0.72, improve diet *p* = 0.72 or see a GP for further assessment *p* = 0.35). At 2 weeks, 32% of participants could recall heart age versus 16% for risk percentage. Heart age recall decreased at 2 weeks (32%) compared with immediately postintervention (65%). Participants with a younger heart age are more likely to recall risk (80% heart age vs. 63% percentage *p* = 0.009*) than those with an older heart age (both 61%; *p* > 0.999*). No difference in format and risk perceptions (*p* = 0.071*). *Mann‐Whitney test
	Lopez‐Gonzalez et al. (2015)	Percentages and control (no risk score)	Reduction in smoking (1.8% heart age vs. 0.4% percentage) and weight (−0.8 kg heart age vs. −0.2 kg percentages) at 12 weeks. At 12 months Framingham risk scores increased in the control group (+0.24%) and decreased in the risk percentage group (−0.2%) and the heart age group (−0.4%).
	Damman et al. (2018)	Percentages and risk ratios	Heart age increased intentions to be more physically active (*F* = 6.29; *p* = 0.13*) and to visit a GP for further screening (*F* = 5.23; *p* = 0.023*). Improved recall of verbal labels (*F* = 7.1; *p* = 0.008*). *ANOVA
Graphical displays			
Icon arrays	Ruiz et al. (2013)	Percentages	Risk recall lower in the icon array group (*p* < 0.001*^1^). No difference in long‐term risk recall (*p* = 0.10*^1^). No difference in risk understanding (*p* = 0.31*^1^). No differences in perceptions of seriousness (*p* = 0.85*^2^), intention to change lifestyle (*p* = 0.15*^2^), intentions to follow medical treatment (*p* = 0.65*^2^) or overall satisfaction (*p* = 0.09*^2^) No differences in clarity (*p* = 0.13*^2^) or helpfulness of information (*p* = 0.43*^2^) *^1^ Chi‐squared test. *^2 ANOVA^
	Zikmund‐Fisher et al. (2014)	Comparison of icons	Risk recall highest in restroom icons and photographs (both 81%). Risk recall lowest in blocks and faces (both 71%).
	Witteman et al. (2014)	Random sequencing	Animated randomness associated with better alignment between risk estimates and risk perceptions (*F*1,3576 = 6.12, *p* = 0.01*) but reduced lifestyle intention scores (*F*1,3572 = 11.1, *p* = 0.01*). Improved risk recall in low‐risk participants (*F*1,3544 = 7.06, *p* = 0.01*). *ANOVA
Icon arrays and bar graphs	French et al. (2004)	Numbers	Participants who received bar graph or icon array had lower levels of worry (*F*1,313–8.74; *p* < 0.01*) but were not more reassured. *ANOVA
	Adarkwah et al. (2019)	N/A each other	No difference in recall of interventions agreed upon with general practitioners at 3 months between the icon array group (1.04 ± 0.44) and the bar graph group (1.05 ± 0.39). Risk perception highest in bar graph group at 3 months (*p* = 0.032)*. Between baseline and 3 months, risk perceptions decreased in the bar graph group (*p* = 0.02)*. There was no change in the icon array group. *Student’s *t* test
	Navar et al. (2018)	N/A each other	22% of participants shown icon array reported a 10‐year risk of 15% to be high compared with 36% shown no icon and 35% shown a bar graph (*p* < 0.001)*. 5%–6% more participants were willing to take preventive treatment when shown bar graph compared with icon array *Two‐tailed test
Avatars	Witteman et al. (2014)	Icons and frequencies	Improved risk perceptions overall (*F*1,13,576 = 4.61, *p* = 0.03). Improved alignment between risk estimates and intentions to see a doctor (*F*1,356 = 6.38, *p* = 0.01) *nested factorial ANOVA
	Ruiz et al. (2016)	Voice and text	Improved intentions to change lifestyle (*p* = 0.3). No differences in risk recall or understanding (χ^2^ = 1.1. *p* = 0.57*). *ANOVA
Qualitative information	Damman et al. (2018)	Infographics	More correct answers for the recall of risk causes when the text was used.
Infographics	Damman et al. (2018)	Qualitative information	Infographics negatively influenced recall of risk causes (*F* = 7.73; *p* = 0.006*). Information evaluated more negatively with infographics (*F* = 8.83; *p* = 0.003*). Infographics negatively influenced subjective risk comprehension (*F* = 10.14; *p* = 0.002*). 67% of participants with adequate health literacy considered infographic information useable versus 54% with inadequate health literacy. Figures rose to 73% and 76% respectively when no graphics were used. *ANOVA
Timeframes			
Lifetime risk	Fair et al. (2008)	10 year risk	Lifetime risk led to higher incidences of worry (*p* = 0.23)*, disturbance (*p* = 0.17)* and less reassurance (*p* = 0.16)*. *ANOVA
5, 10, 15 & 20 year risk	Frileux et al. (2004)	N/A each other	Shorter timeframes led to higher intentions to adopt preventive behaviours (*F*3,414 = 229.33: *p* < 0.00001). *ANOVA with repeated measures
Genetic Risk Scores	Domenech et al. (2016)	No risk score	18 (58.1%) participants in the genetic risk score group had hypertension control compared with 14 (38.9%) in the control group (*p* = 0.008)* at 16 weeks *Pearson chi‐square test
	Knowles et al. (2017)	Framingham risk score	No difference in low‐density lipoprotein cholesterol at 3 months (*p* = 0.59)* or at 6 months (*p* = 0.75)*. The genetic risk score group reported moderate weight loss in high‐risk participants (−2.3 kg ± 3 vs. 0.0 kg ± 3, *p* = 0.002*). *Hodges‐Lehmann statistic
Cardiovascular imaging			
Coronary artery calcium scores	Johnson et al. (2015)	No comparison	68% of participants could accurately identify their risk score based on their coronary artery calcium score. 24% of high‐risk participants identified that they were in the high‐risk group. There were improvements in health‐promoting behaviour (*p* < 0.001*). *ANOVA
	Orakzai et al. (2008)	No comparison	Initiating aspirin therapy, increasing exercise and modifying diet increased with increasing coronary artery calcium scores (all *p* < 0.001* for trends). 56% high‐risk participants modified their diet and 67% increased exercise. *t test and Mann‐Whitney rank‐sum test
	Kalia et al. (2006)	No comparison	Statin compliance at 3 (±2) years was highest in the group with the highest coronary artery calcium scores (91%) and lowest in the low‐risk group (44%). Dietary modifications increased from 41% to 64%. 71% stopped smoking. 65% increased exercise.
Carotid ultrasounds	Näslund et al. (2019)	Percentages	Largest decrease in Framingham risk scores in the carotid visualization group (−0∙58 [95% CI –0∙86 to −0∙30] vs. 0∙35 [0∙08–0∙63]). Larger reduction in low‐density lipoprotein cholesterol in the carotid visualization group (0.3 mmol/L vs. 0.12 mmol/L). Larger decrease in smoking in carotid visualization group (1.25% vs. 1.01%).
	Korcarz et al. (2008)	No comparison	Higher levels of plaque led to increased intentions to take cholesterol‐lowering medication (*p* = 0.02*) and an increased likelihood of having heart disease (*p* = 0.004*) and developing heart disease (*p* < 0.001*). Normal scans also lead to increased motivation to exercise (*p* = 0.003*). *multiple linear regression model

**TABLE 4:** Perceptions of risk communication strategy

Risk communication strategy	Participant quotes
Numerical formats	
Numbers	‘Going to make me go online or make an appointment with a doctor who can make it clearer’ (Ancker et al., 2009)
Percentages	‘oh that’s only half of the risk! Let’s take a look…your risk is 42%. Then it could have been worse’ (Damman et al., 2016) ‘I have 2%...what does that mean…does that mean 2 days out of 100 I'm at risk?’ (Bonner et al., 2014)
Heart age	‘I hate this 74 and 72, that's not real… The only one who can say what my heart age would be is the cardiologist when he goes in and has a look at my heart’ (Bonner, Jansen, McKinn, et al., 2014; Bonner, Jansen, Newell, et al., 2014) ‘I mean, I already feel that I am healthy‐ish for my age.. to me that says yeah you’re ok’ (Bonner, Jansen, McKinn, et al., 2014; Bonner, Jansen, Newell, et al., 2014) ‘Wow this is very good…It's an eye‐opener… oh yeah I'm overweight and this and that but never thinking that it (would) have such an impact on my heart’ (Bonner, Jansen, McKinn, et al., 2014; Bonner, Jansen, Newell, et al., 2014) ‘I'm thinking that it's kind of overwhelming. It's intimidating for a man to come in who is 52 and find out he's got a heart age of 79. I think it's going to be very upsetting. He’s gonna be really shaken’ (Goldman et al., 2006) ‘I think the idea of [cardiovascular risk‐adjusted age] made it personal. Because this is your age. It brought you into it’ (Goldman et al., 2006)
Graphical formats	
Bar graphs	‘well I’m not above the 50%, I'm in the red zone but the lower part of it’ (Damman et al., 2016)
Icon arrays	‘It's a lot to look at’ (Ancker et al., 2009) ‘clearer that you’re talking about human beings and not statistics’ (Ancker et al., 2009) ‘It can give a false reading’ (participant talking about random sequencing) (Ancker et al., 2009)
Game interaction	‘It's like a game because you’re playing around with it. That's what I like about it because you learn too’ (Ancker et al., 2009)
Genetic Risk Score	‘if you have a high genetic risk it's in your genes… deprived yourself of all your nice treats but you’ve had the same end result, you might as well have enjoyed it and gone!’ (Shefer et al., 2016) ‘If it's going to run in the family you’ve got to accept it haven'’t you? If it's your turn to, if your number comes up you can’t do nothing about it’ (Middlemass et al., 2014) ‘I was sure there was something in the family make up but it's nice to know that's not the case’ (Middlemass et al., 2014) ‘The lifestyle I have led puts me at a greater risk than the person who didn’t live my lifestyle’ (Middlemass et al., 2014)

### 2.5 Quality appraisal

A quality assessment was undertaken for included studies by one reviewer (SS) and a second reviewer (AF) appraised 30% of the studies to ensure consistency (supplementary material online). The quality appraisal was based on criteria from the Critical Appraisal Skills Programme (CASP) tools for qualitative, randomized controlled trials, case‐controlled studies and cohort studies.

### 2.6 Data abstraction

Separate quantitative and qualitative data extraction forms were developed to collect data from eligible studies and included: location, study design, participant characteristics, data collection methods, risk communication strategy and outcome data. The data were then visually presented in a table.

### 2.7 Data synthesis

Due to the heterogeneity in study designs and outcomes, a meta‐analysis was not feasible. A narrative synthesis, which adopts a textual approach to summarize the findings of systematic reviews, was performed for the quantitative studies in accordance with the Economic and Social Research Council (ESRC) methods programme guidance on narrative synthesis in systematic reviews (Popay et al., 2006). The data extraction forms were used to produce a descriptive summary, organizing data by communication strategy. The study outcomes and results were then tabulated (Table 2). A narrative summary (Dixon‐Woods et al., 2004) was undertaken for the qualitative studies, and the data were summarized under the same categories. The quantitative and qualitative results were integrated and the reviewers used concept mapping to explore relationships by producing a commentary of the data.

## 3 RESULTS

### 3.1 Summary of findings

**FIGURE 2:**
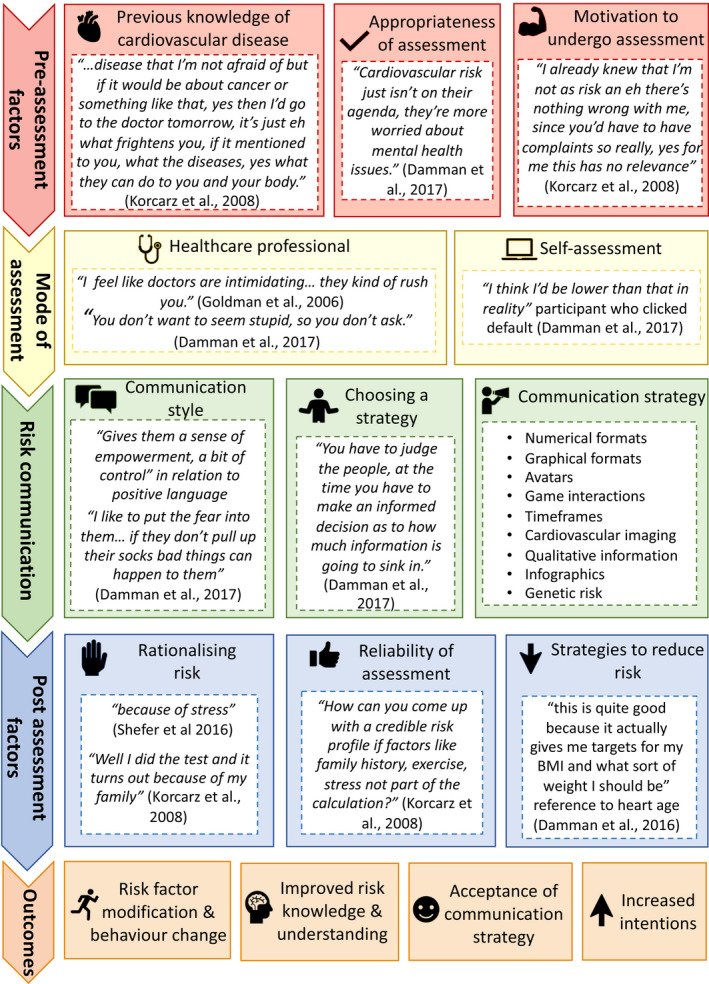
Factors during cardiovascular assessment that influence risk communication.

Nine different categories of cardiovascular risk communication strategies were identified and summarized under the following headings: numerical formats, graphical formats, qualitative information, infographics, avatars, game interactions, timeframes, genetic risk scores and cardiovascular imaging. In addition to these, the analysis of the qualitative studies identified multiple factors which may also influence risk communication. These have been categorized into pre‐assessment factors, mode of assessment, risk communication and post‐assessment (Figure 2 and Table S4).

### 3.2 Study quality

Two of the qualitative studies scored low overall using the CASP risk appraisal tool and the remaining studies were of medium quality. The components with the poorest scores were rigour of data analysis, clarity of statement of findings and design. Two of the studies (Bonner, Jansen, McKinn, et al., 2014; Bonner, Jansen, Newell, et al., 2014; Hill et al., 2010) failed to describe the qualitative approach taken. Overall, most of the quantitative studies addressed a clearly focussed issue, considered all important outcomes and had results in keeping with existing evidence. Studies scored lower in recruitment strategies and generalizability of the results. This was attributable to several biases including recruitment of a majority or all‐male sample (Powers et al., 2011; Ruiz et al., 2013, 2016) and participants at low cardiovascular risk (Bonner et al., 2015; Lopez‐Gonzalez et al., 2015). A small proportion of studies scored low in minimizing bias while measuring outcomes (French et al., 2004) and reasons include using research‐developed tools as opposed to validated ones (Adarkwah et al., 2019).

### 3.3 Risk communication strategies

#### 3.3.1 Numerical formats

This describes quantitative risk information provided as percentages, risk ratios or heart age scores and was investigated in eight studies (five quantitative and three qualitative). In a randomized trial, participants were more likely to agree that risk information was presented clearly and more helpful and reported less decisional conflict in choosing their preferred risk reduction method when presented with their Framingham risk score as opposed to risk factor education only (Powers et al., 2011). There were no differences in health behaviours, blood pressure or medication adherence and perceived risk declined in both groups at 3 months.

Damman et al. (2016) identified that providing estimates of the percentage of similar individuals who will have a cardiovascular event at a given time period failed to heighten risk perception as some participants believed a risk score below 50% implied low risk. Participants also had problems recalling percentages, especially when provided with multiple numbers from their assessment. This was reiterated in a second qualitative study (Bonner, Jansen, McKinn, et al., 2014; Bonner, Jansen, Newell, et al., 2014), where participants had difficulties remembering and understanding their risk percentage.

Only one quantitative study (Fair et al., 2008) investigated risk ratios by comparing them to percentages. Risk ratios increased risk perceptions and intentions to make lifestyle changes, however, also increased levels of worry.

The final numerical strategy evaluated was the use of an estimated heart age, which calculates an individual's heart age based on their risk profile, and was investigated in three quantitative studies. Bonner et al. (2015) compared the effect of providing individuals with their heart age against a percentage event rate at 5 years on behavioural and psychological outcomes. There was no significant difference in intention to change lifestyle or in risk perceptions. At 2 weeks, recall was highest in the heart age group but had significantly decreased since the intervention. Those with a younger heart age were more likely to recall their risk than those in the percentage group, however, there was no difference in recall between groups in participants with an older heart age. Participants in the heart age group, however, perceived the results to be less credible and had less of a positive emotional response.

Another study (Lopez‐Gonzalez et al., 2015) compared the effect of the heart age on modifiable cardiovascular risk factors against a percentage event rate and a control group, who received conventional medical advice only. At 12 weeks, there was a significant decrease in weight and smoking in both experimental groups compared with the control group but was accentuated in the heart age group. At 12 months, Framingham risk scores had increased in the control group but decreased in the heart age and percentage groups. When the heart age was compared with either a percentage or natural frequency, the heart age improved intentions to become more physically active and to visit a general practitioner (Damman et al., 2018).

Two qualitative studies addressed the acceptability of the heart age. High‐risk participants were less accepting of their results and questioned their credibility (Bonner, Jansen, McKinn, et al., 2014; Bonner, Jansen, Newell, et al., 2014). Those with a heart age that closely reflected their own age were also more reassured. In the study by Goldman et al. (2006), participants felt that the heart age score would be more memorable, but warned that receiving an older heart age may increase anxiety in individuals. A further qualitative study (Ancker et al., 2009) revealed that although some individuals were accepting numerical risk information, others found it too impersonal.

#### 3.3.2 Graphical formats

The risk was visually represented in graphical formats in eight studies (six quantitative and two qualitative) including bar graphs and icon arrays. Risk recall was lower when presented as an icon array compared with a numerical format immediately post‐intervention, with no differences between the groups at 2–3 weeks on recall or understanding of risk (Ruiz et al., 2013). There were also no significant differences in perceptions of seriousness, intentions to change lifestyle, follow medical treatment or overall satisfaction. Moreover, there were no differences in clarity or helpfulness of the information between the groups. Additionally, participants who received their risk in a graphical format (bar graph or icon array) compared with a numerical format (percentage and risk ratio) reported lower levels of worry but were not more reassured (French et al., 2004).

In a randomized trial, where icon arrays were compared directly with bar graphs (Adarkwah et al., 2019), no differences were observed in the recall of interventions agreed on with a general practitioner at 3 months. Risk perceptions were highest in the bar graph group at 3 months but decreased in comparison with baseline, whereas it remained consistent in the icon array group. Additionally, in a second study, participants who were shown their risk as a bar graph had a higher perceived risk and were more likely to take preventative treatment than those who received an icon array (Navar et al., 2018). Conversely, a qualitative study (Damman et al., 2016) highlighted individuals may misinterpret the severity of their results when using bar graphs because high scores such as 20% appear to be in the lower portion of the graph.

The type of icon used also influenced perceptions and risk recall. The icons which performed better in risk recall were restroom icons and photographs with blocks and faces performing the worst (Zikmund‐Fisher et al., 2014). Mean perceived cardiovascular risk perceptions did not significantly differ and were moderately correlated with the actual risk information that was presented. Random rather than sequential positioning of negative icons to portray the chance of suffering a cardiovascular event was associated with better alignment between risk estimates and perceptions but reduced lifestyle intention scores (Witteman et al., 2014). Random dispersal was also reported as more realistic in a qualitative study. (Ancker et al., 2009). Participants found icon arrays with stick icons more personal and relatable than those which use shapes.

#### 3.3.3 Avatars

Avatars are digital representations of people used to promote social interaction (Ruiz et al., 2016) and were addressed in two quantitative studies. Avatars improved overall risk perceptions among participants and alignments between risk estimates and intentions to see a doctor (Witteman et al., 2014). Conversely, no differences in risk recall or understanding were found when avatars were compared against voice and text alone (Ruiz et al., 2016). There were no significant differences in worry, disturbance or confidence to follow medical treatments, however, the avatar was favoured in intentions to make lifestyle changes.

#### 3.3.4 Qualitative information

Qualitative information can be provided to individuals to help structure how a lay person thinks about risk. Qualitative information was investigated in one quantitative study. Participants were more likely to provide correct answers for risk recall and subjective risk comprehension questions when qualitative information was used to communicate risk compared with infographics.

#### 3.3.5 Infographics

Infographics are sophisticated visualizations compromised of imagery, charts and text to provide an overview of a topic. They also provide additional narratives such as information about risk factors. One quantitative study investigated the use of infographics and found that infographics negatively influenced the recall of risk causes and subjective risk comprehension and more correct answers were given when qualitative information was used (Damman et al., 2018). Health literacy also influenced results as participants with adequate health literacy were more likely to consider infographic information useful.

#### 3.3.6 Game interactions

Game‐like interactions are those which permit individuals to interact with the information provided to them (Ancker et al., 2009). Only one qualitative study (Ancker et al., 2009) investigated the use of an interactive game format to portray cardiovascular risk and involved clicking different icons to reveal which individuals would be affected by cardiovascular disease. Some participants enjoyed the interactive component which made it more like a game, whereas others found the process time‐consuming.

#### 3.3.7 Timeframes

Time‐based risk formats allow for timeframe manipulations and can portray the accrual of risk over time. Timeframes were addressed in two quantitative studies. Participants who were shown their lifetime cardiovascular risk reported higher incidences of worry and were less reassured than participants who received a 10‐year cardiovascular risk (Fair et al., 2008). Similarly, in a study (Frileux et al., 2004), where five different timeframes (ranging from 5 to 20 years) were investigated, the shorter timeframes performed better. Higher intentions to adopt preventative behaviour were also reported when shorter timeframes were used.

#### 3.3.8 Genetic risk scores

Genetic risk scores use statistical measures of genome variations that increase an individual’s probability of developing cardiovascular disease (Shefer et al., 2016). Four studies (two quantitative and two qualitative) investigated the use of providing individuals with feedback from a genetic risk score to communicate cardiovascular risk. Receiving a genetic risk score was associated with improved hypertension control at 16 weeks compared with participants who received no risk information in a randomized controlled trial. (Domenech et al., 2016). When comparing the Framingham risk score only to the Framingham risk score plus genetic risk score, there was no effect on low‐density lipoprotein cholesterol at 3 or 6 months. High‐risk participants in the genetic risk score group did, however, report a moderate loss in weight (Knowles et al., 2017).

Shefer et al. (2016) identified that when participants were provided with both their Framingham risk score and genetic risk score, they often only remembered one score and were unable to recall which one it was. Furthermore, participants often misunderstood their genetic risk score, believing if their risk was ‘in their genes’ it could not be modified. This misconception was also highlighted in a second qualitative study (Middlemass et al., 2014). One participant, however, recognized the importance of the gene and environment interaction and that lifestyle modification could reduce an increased genetic risk. Participants with a low genetic risk score felt reassured by their results, particularly those with a family history of cardiovascular disease (Middlemass et al., 2014).

#### 3.3.9 Cardiovascular imaging

The results from cardiovascular imaging, including coronary artery calcium scoring and carotid ultrasound measurements, can be used to provide feedback to individuals about their current cardiovascular health and risk of a future event. Cardiovascular imaging was addressed in five quantitative studies. Three quantitative studies investigated the use of coronary artery calcium scores. The first (Johnson et al., 2015) provided participants with their coronary artery calcium score and verbal risk category. Overall, over two‐thirds of the participants could accurately identify their risk category based on their coronary artery calcium score. A significantly lower proportion of high‐risk participants identified that their score placed them in the high‐risk category. All five risk groups showed improvements in health‐promoting behaviour; however, there were no changes in risk perceptions over time. Worry was highest in the low‐risk group at baseline but highest in the moderate‐ and high‐risk groups at 3 months. In the other two studies, participants were provided with verbal information about their coronary artery calcium scores. The studies were similar in design and may include the same participants but reported different outcomes. Orakzai et al. (2008) determined that the number of participants who reported initiating aspirin therapy, increasing exercise and modifying their diet increased with increasing coronary artery calcium scores. Over half of the participants in the highest risk category reported modifying their diet and increasing exercise. In the second study (Kalia et al., 2006), statin compliance at 3 years was highest in participants with high coronary artery calcium scores and lowest in those with low‐risk scores. Overall, a large proportion of participants reported increasing exercise levels, stopping smoking and making dietary modifications.

In two studies, participants were provided with the results of their carotid ultrasound scan. In a randomized controlled trial, Framingham risk scores decreased at 1 year among participants who received a visualization of their scan result and increased in the group who received a risk score only (Näslund et al., 2019). Additionally, there was a greater reduction in low‐density lipoprotein cholesterol concentrations and smoking rates in the group who received a visualization of their carotid ultrasound. Korcarz et al. (2008) provided participants with verbal information about their carotid ultrasound. Participants with increased levels of plaque reported increased perceptions of having or developing heart disease and intentions to take cholesterol‐lowering medication.

### 3.4 Factors that impact on cardiovascular risk communication

#### 3.4.1 Pre‐assessment factors

Three pre‐assessment factors were identified from the review including previous knowledge of cardiovascular disease. Some participants did not understand what cardiovascular disease encompassed and it was perceived as less frightening than cancer (Damman et al., 2017). Some believed that they had adequate knowledge of cardiovascular disease and risk factors, deeming themselves not at risk. This links to the second factor, which is motivation to undergo cardiovascular risk assessment. Some participants who believed that they were of low risk were unlikely to undergo screening, particularly when they had no physical complaints (Damman et al., 2017). The appropriateness of a cardiovascular assessment was also highlighted by healthcare professionals. They believed that if patients had more pressing health concerns, discussing their cardiovascular risk would place an additional burden on them (Bonner, Jansen, McKinn, et al., 2014; Bonner, Jansen, Newell, et al., 2014).

#### 3.4.2 Mode of assessment

Self‐assessment and assessment by healthcare professionals were the two modes of cardiovascular assessment identified. Some participants felt that their doctor was too busy or intimidating to conduct an assessment (Ancker et al., 2009). They were also too embarrassed to request clarification if they did not comprehend their risk (Ancker et al., 2009). Online calculators allowed individuals to conduct their own assessment, but many underestimated their risk factor values and entered incorrect data (Bonner, Jansen, McKinn, et al., 2014; Bonner, Jansen, Newell, et al., 2014). Furthermore, if they did not understand their risk, they were unable to seek clarification from a healthcare professional.

#### 3.4.3 Communication of risk

As well as choosing which communication strategy to use, healthcare professionals highlighted that they also use different communication styles. Some opt for paternalistic communication styles promoting fear, whereas others prefer to empower patients (Bonner, Jansen, McKinn, et al., 2014; Bonner, Jansen, Newell, et al., 2014). Furthermore, they identified that they chose which strategy to use based on how much information they believed patients could process. For example, they believed that colourful charts were most beneficial in patients with low health literacy (Bonner, Jansen, McKinn, et al., 2014; Bonner, Jansen, Newell, et al., 2014).

#### 3.4.4 Post‐assessment

Three post‐assessment factors were identified including perceived reliability of assessment, rationalizing risk and reducing risk. Participants questioned the reliability of some probabilistic risk scores because they expected to provide more information about their health and behaviour (Damman et al., 2016). Many went on to rationalize their risk, blaming factors out of their control including stress or family history (Damman et al., 2016). One participant felt that the heart age score encouraged risk reduction as it provided a target to work towards (Bonner, Jansen, McKinn, et al., 2014; Bonner, Jansen, Newell, et al., 2014). Another participant identified that they would be more willing to take risk reduction measures if they had decided on them in partnership with their doctor (Sheridan et al., 2009).

## 4 DISCUSSION

This is the first systematic review of both quantitative and qualitative studies into cardiovascular risk communication strategies. Nine different categories of cardiovascular risk communication strategies were identified. The results reveal that multiple factors are involved in communicating cardiovascular risk which influence acceptance, risk understanding and health factor modification. We report that strategies providing the results of cardiovascular imaging and an estimation of heart age were the most effective at communicating cardiovascular risk. Conversely, those attempting to represent risk using bar charts, percentages and infographics were less successful.

Cardiovascular imaging feedback and estimates of heart health are both methods of portraying personalized risk information. This is more relatable and therefore enhances risk perceptions and drives behaviour change. This aligns with research conducted by Lawton (2002) which proposes that health behaviour change is reactive and not proactive. Providing individuals with physical evidence of cardiovascular diseases, such as is demonstrated on the scan or with a heart age above that of the actual age, may provide a cue to action. The heart age is more memorable and understandable because it conveys to individuals how suboptimal their heart is.

Individuals are less engaged in risk discussions when physical symptoms are absent, with many unaware symptoms of cardiovascular disease are not present until the cardiovascular disease is advanced. Cardiovascular imaging removes any uncertainties with the notion of risk by providing direct evidence of cardiovascular disease. This is in keeping with previous research where strategies which employed imaging or visual techniques were the most effective at communicating personalized risk (French et al., 2017). It is also important to consider the limitations associated with using cardiovascular imaging to communicate cardiovascular risk. Imaging is more time‐consuming and expensive than traditional methods and imaging technologies may not be readily available or accessible in developing countries. Moreover, it may provide false reassurance as normal scans in young individuals with unhealthy lifestyles may incorrectly suggest that they can continue with their current lifestyle.

Several risk communication strategies, such as percentages, bar graphs and icon arrays, which provide patients with a probability, fail to heighten risk perceptions (Damman et al., 2016). Many of the most commonly used cardiovascular risk scores, such as the Framingham score, classify a 20% risk of developing cardiovascular disease over the next 10 years as high. As 20% appears in the lower portion of the graphs, these scores can be interpreted as low risk. The same is true for icon arrays as a large number of positive icons makes it easy for participants to believe that they would be one of the individuals not affected (Ancker et al., 2009).

The genetic risk score also provides individuals with a probabilistic risk score. One study claimed that providing a genetic risk score improved blood pressure control (Domenech et al., 2016). It is difficult to establish if this was the result of receiving a genetic risk score or an improved awareness of risk because the control group received no risk information. Participants believed that their genetic risk could not be modified and consequently were less motivated to make lifestyle improvements. The genetic risk assessment and gene and lifestyle interaction require more explanation to individuals.

In addition to the communication strategy, it was identified that a wider range of factors can influence the success of cardiovascular risk communication. These factors may affect how well a particular strategy works and highlights individuals may perceive risk differently based on their values, environment and relationship with a health professional. This is in keeping with the first two components of de Haes and Bensing’s medical communication model (de Haes & Bensing, 2009). The first element involves fostering a relationship with patients. Healthcare professionals must ensure an open and transparent relationship, where patients feel empowered to ask questions. Second, before providing risk information professionals should gather information from patients, enabling them to understand patient beliefs and values and choose the best communication strategy. The other components of the model include decision‐making, enabling disease and treatment‐related behaviour and responding to emotions.

Responding to emotions is also crucial and those involved in risk discussions must recognize the potential negative impact of being identified as high risk. Those designated as being at risk who previously viewed themselves as healthy are now faced with a revised health status despite lacking the associated symptoms (Gillespie, 2015). This may bring new social manifestations and health regimes similar to those who are ill. Care must be taken when broaching high‐risk discussions and professionals must offer suitable support.

### 4.1 Strengths and limitations

This is the first systematic review of quantitative and qualitative studies into cardiovascular risk communication strategies and included a large number of studies from a range of countries. The narrative synthesis approach enabled an overview of the large and diverse evidence base. As this review involved both quantitative and qualitative studies, we were able to expand on the quantitative results by identifying other factors which influence the success of cardiovascular risk communication.

It is, however, important to address the limitations associated with this review. First, narrative synthesis analysis is open to subjective interpretation. However, the inclusion of tabulated data helps ensure transparency. The studies were conducted in high‐income western countries and the cultural bias of the investigators may have influenced their lens of inquiry and analysis. In addition, despite no language restrictions, all studies were published in English, which may have excluded important cultural contexts. This may affect the generalizability of the results, as individuals’ cultural backgrounds and beliefs impact their perceptions of healthcare and lifestyle practices. Another limiting factor is that some of the studies provided participants with hypothetical risk scenarios, which may not have elicited true emotional responses. In addition, some of the studies included participants with low cardiovascular risk or those interested in their health. It is also possible that partaking in research led to participants overestimating health behaviours particularly in the self‐reported data. Some of the included risk communication strategies could be considered complex interventions and it can be difficult to unpick the active ingredient with the success dependent on many factors. Healthcare professionals must consider the optimization of the communication strategy in their setting.

## 5 CONCLUSION

The cardiovascular risk communication strategy which had the most significant impact on cardiovascular health behaviours and risk factors were those which used cardiovascular imaging. More high‐quality randomized controlled trials are required to confirm these findings and determine the effect of these strategies on long‐term behaviour change and other important clinical outcomes such as reduced cardiovascular mortality. Qualitative studies into individuals’ perceptions of cardiovascular imaging risk communication strategies would also help to identify the key components contributing to its benefits as a risk communication strategy. Our findings, however, suggest that future risk communication strategies would be more successful if they incorporated methods to provide individuals with evidence of their current cardiovascular health status.

## AUTHORS’ CONTRIBUTION

SS, NLM, FS and LN conceived the study and its design. SS, FS, KJ and AF conducted the searches and performed the screening. SS and AF performed the quality assessment for included studies. SS acquired and interpreted the data. AF, FS and LM provided feedback on the qualitative analysis. SS, NLM, LN and FS drafted the manuscript. All authors have agreed on the final version and meet at least one of the following criteria (recommended by the ICMJE: http://www.icmje.org/recommendations/):

1. substantial contributions to conception and design, acquisition of data or analysis and interpretation of data;
2. drafting the article or revising it critically for important intellectual content.

## FUNDING INFORMATION

This study was funded by a British Heart Foundation Research Training Fellowship for Nurses and Healthcare Professionals Clinical (FS/RTF/21/30028) awarded to SS. NLM is supported by a Chair Award, Programme Grant and Research Excellence Award (CH/F/21/90010, RG/20/10/34966 and RE/18/5/34216) from the British Heart Foundation.

## CONFLICT OF INTEREST

No conflict of interest has been declared by the author(s).

### PEER REVIEW

The peer review history for this article is available at https://publons.com/publon/10.1111/jan.15327.

## Supporting information


**Appendix S1.** Xxxx

**Appendix S2.** Xxxx

**Appendix S3.** Xxxx

## Data Availability

Data sharing is not applicable to this article as no new data were created or analyzed in this study.
