# Supplementary material for: Cardiovascular risk communication strategies in primary prevention. A systematic review with narrative synthesis
Source: J Adv Nurs. 2022 Jun 19;78(10):3116–40. doi: 10.1111/jan.15327 (PMC9546276; doi:10.1111/jan.15327)
Supplement: Supplementary file 1 — Appendix S1. Xxxx [file JAN-78-3116-s002.docx]

**Supplementary Data**

**Search Terms**

**CINAHL**

1. (MH "Cardiovascular Diseases/RF/PC/ED/DI")
2. (MH "Cardiovascular Risk Factors")
3. "cardiovascular"
4. "cardiac"
5. (MH "Coronary Arteriosclerosis")
6. "coronary artery disease"
7. "coronary heart disease"
8. (MH "Coronary Disease")
9. "heart disease" OR (MH "Heart Diseases")
10. (MH "Atherosclerosis")
11. (MH "Myocardial Ischemia")
12. "cardiovascular prevention"
13. "ischaemic heart disease"
14. "cardiovascular primary prevention"
15. S1 OR S2 OR S3 OR S4 OR S5 OR S6 OR S7 OR S8 OR S9 OR S10 OR S11 OR S12 OR S13 OR S14
16. "risk communication"
17. "risk format"
18. (MH "Risk Assessment")
19. "risk assessment tools"
20. "risk messages"
21. "risk score"
22. "risk presentation"
23. "presentation format"
24. "risk communication strategies"
25. "decision aids"
26. "SCORE"
27. "framingham risk score"
28. "ASSIGN"
29. "risk communication tool"
30. "QRISK"
31. "risk calculator"
32. "risk prediction chart"
33. "risk representation"
34. S16 OR S17 OR S18 OR S19 OR S20 OR S21 OR S22 OR S23 OR S24 OR S25 OR S26 OR S27 OR S28 OR S29 OR S30 OR S31 OR S32 OR S33
35. (MH "Behavioral Changes")
36. (MH "Health Behavior")
37. "health behavior change"
38. (MH "Behavior Modification")
39. "risk factor modification"
40. (MH "Risk Taking Behavior")
41. (MH "Attitude to Risk")
42. "risk reduction"
43. (MH "Life Style Changes")
44. "lifestyle modification"
45. "lifestyle choices"
46. (MH "Perception")
47. (MH "Attitude")
48. "opinion"
49. "understanding"
50. "risk modification"
51. "experiences"
52. "acceptability"
53. (MH "Self Care")
54. S35 OR S36 OR S37 OR S38 OR S39 OR S40 OR S41 OR S42 OR S43 OR S44 OR S45 OR S46 OR S47 OR S48 OR S49 OR S50 OR S51 OR S52 OR S53
55. S15 AND S34 AND S54

**Ovid EMBASE**

1. coronary artery disease/
2. ischemic heart disease/
3. coronary heart disease.mp.
4. heart disease/
5. cardiovascular primary prevention.mp.
6. atherosclerosis/
7. cardiovascular risk factors.mp.
8. risk communication.mp.
9. risk tools.mp.
10. decision aids.mp.
11. risk presentation.mp.
12. risk messages.mp.
13. risk score.mp.
14. risk assessment/
15. risk format.mp.
16. presentation formats.mp.
17. risk representation.mp.
18. risk communication strategies.mp.
19. risk communication tool.mp.
20. ASSIGN.mp.
21. QRISK score/
22. heart age.mp.
23. risk presentation charts.mp.
24. risk calculator.mp.
25. risk assessment tools.mp.
26. health behaviour change.mp.
27. risk factor modification.mp.
28. risk reduction/
29. acceptability.mp.
30. experience/
31. behavior change/
32. lifestyle change.mp. or lifestyle modification/
33. risk modification.mp.
34. self care/
35. perception/
36. comprehension/
37. health behavior/
38. behavior modification/
39. risk taking behavior.mp.
40. attitude to risk.mp.
41. lifestyle choices.mp.
42. attitude/
43. cardiovascular disease/di, ep, pc [Diagnosis,

Epidemiology, Prevention]

1. cardiovascular risk/
2. 1 or 2 or 3 or 4 or 5 or 6 or 7 or 43 or 44
3. 8 or 9 or 10 or 11 or 12 or 13 or 14 or 15 or 16 or 17 or

18 or 19 or 20 or 21 or 22 or 23 or 24 or 25

1. 26 or 27 or 28 or 29 or 30 or 31 or 32 or 33 or 34 or 35 or

36 or 37 or 38 or 39 or 40 or 41 or 42

1. 45 and 46 and 47

**Ovid Medline**

1. coronary heart disease.mp.
2. Coronary Artery Disease/
3. Heart Diseases/
4. cardiac.mp.
5. cardiovascular primary prevention.mp.
6. Atherosclerosis/
7. cardiovascular risk factors.mp.
8. Myocardial Ischemia/ or ischaemic heart disease.mp. 43602 Advanced
9. cardiovascular prevention.mp.
10. risk communication.mp.
11. risk tools.mp.
12. decision aids.mp.
13. risk presentation.mp.
14. risk messages.mp.
15. risk score.mp.
16. Risk Assessment/
17. risk reduction.mp.
18. risk format.mp.
19. presentation formats.mp.
20. risk representation.mp.
21. risk communication strategy.mp.
22. Communication/
23. risk communication tool.mp.
24. ASSIGN.mp.
25. Heart Age.mp.
26. QRISK.mp.
27. risk presentation charts.mp.
28. risk calculator.mp.
29. risk assessment tools.mp.
30. health behav* change.mp.
31. risk factor modification.mp.
32. risk reduction.mp. or Risk Reduction Behavior/
33. accept*.mp.
34. Health Behavior/ or behavior change.mp.
35. lifestyle change.mp.
36. risk modification.mp.
37. Self Care/
38. Perception/
39. behav* modification.mp.
40. risk taking behav*.mp.
41. Health Knowledge, Attitudes, Practice/ or attitude to risk.mp.
42. Life Style/ or lifestyle modification.mp.
43. Attitude to Health/ or Attitude/
44. lifestyle change.mp.
45. Comprehension/
46. cardiovascular.mp.
47. Coronary Artery Disease/dh, pc, px [Diet Therapy, Prevention & Control, Psychology]
48. Cardiovascular Diseases/dh, pc, px [Diet Therapy, Prevention & Control, Psychology]
49. Coronary Disease/dh, pc, px [Diet Therapy, Prevention & Control, Psychology]
50. experience.mp.
51. 10 or 11 or 12 or 13 or 14 or 15 or 16 or 17 or 18 or 19 or 20 or 21 or 22 or 23 or 24 or 25 or 26 or 27 or 28 or 29
52. 30 or 31 or 32 or 33 or 34 or 35 or 36 or 37 or 38 or 39 or 40 or 41 or 42 or 43 or 44 or 45 or 50
53. 1 or 2 or 3 or 5 or 6 or 7 or 8 or 9 or 10 or 47 or 48 or 49
54. 51 and 52 and 53

**Web of Science**

1. Cardiovascular
2. "cardiovascular disease"
3. "coronary disease"
4. "coronary heart disease"
5. “coronary artery disease”
6. "heart disease"
7. "cardiovascular primary prevention"
8. Atherosclerosis
9. “cardiovascular risk factors”
10. "Ischemic heart disease"
11. “cardiovascular prevention”
12. #11 OR #10 OR #9 OR #8 OR #7 OR #6 OR #5 OR #4 OR #3 OR #2 OR #1
13. "risk communication"
14. "risk tools"
15. "Decision aids"
16. "risk presentation"
17. "risk messages"
18. "risk score"
19. "risk assessment"
20. "risk format"
21. "presentation formats"
22. "risk representation"
23. "risk communication strategy"
24. "risk communication strategies"
25. "risk communication tool"
26. ASSIGN
27. "Heart age"
28. QRISK
29. "Risk presentation charts"
30. "risk calculator"
31. "risk assessment tools"
32. Attitude
33. "lifestyle choices"
34. "lifestyle modification"
35. "attitude to risk"
36. "risk taking behav*"
37. “Behav* modification”
38. "Health behav*"
39. Understanding
40. Perception
41. "self-care"
42. "risk modification"
43. "Lifestyle chang*"
44. "Behav* chang*"
45. accept*
46. acceptability
47. "risk reduction"
48. "risk factor modification"
49. "Health behav* change"
50. #49 OR #48 OR #47 OR #46 OR #45 OR #44 OR #43 OR #42 OR #41 OR #40 OR #39 OR #38 OR #37 OR #36 OR #35 OR #34 OR #33 OR #32
51. #31 OR #30 OR #29 OR #28 OR #27 OR #26 OR #25 OR #24 OR #23 OR #22 OR #21 OR #20 OR #19 OR #18 OR #17 OR #16 OR #15 OR #14 OR #13
52. #51 AND #50 AND #12

**ASSIA (ProQuest)**

1. Cardiovascular
2. cardiovascular disease
3. coronary disease
4. coronary heart disease
5. coronary artery disease
6. heart disease
7. cardiac
8. cardiovascular primary prevention
9. atherosclerosis
10. cardiovascular risk factors
11. ischemic heart disease
12. cardiovascular prevention
13. risk communication
14. risk tools
15. decision aids
16. risk presentation
17. risk messages
18. risk score
19. risk assessment
20. risk format
21. presentation formats
22. risk representation
23. risk communication strategy
24. risk communication tool
25. ASSIGN
26. SCORE
27. Heart Age
28. QRISK
29. Risk presentation charts
30. Risk calculator
31. Risk assessment tool
32. Attitude
33. Lifestyle choices
34. Lifestyle modification
35. Attitude to risk
36. Risk taking behaviour
37. Behaviour modification
38. Health behaviour
39. Health behaviour change
40. Risk factor modification
41. Risk reduction
42. Acceptability
43. Experiences
44. Behaviour change
45. Lifestyle change
46. Risk modification
47. Self care
48. Perception
49. Understanding
50. 49 or 48 or 47 or 46 or 45 or 44 or 43 or 42 or 41 or 40 or 39 or 38 or 37 or 36 or 35 or 34 or 33 or 32
51. 31 or 30 or 29 or 28 or 27 or 26 or 25 or 24 or 23 or 22 or 21 or 20 or 19 or 18 or 17 or 16 or 15 or 14 or 13
52. 1 or 2 or 3 or 4 or 5 or 6 or 7 or 8 or 9 or 10 or 11 or 12
53. 52 AND 51 AND 50

**Table S1 : Population, Intervention Comparison and Study Design (PICOS) Criteria**

| Population | Adults without established cardiovascular disease |
| --- | --- |
| Intervention | Cardiovascular risk communication strategy |
| Comparison | Routine care or another cardiovascular risk communication approach |
| Outcome | Risk understanding, acceptability of strategy, intention to change health behaviour or risk factor modification/ behaviour health change |
| Study Design | Randomised controlled trials, cohort studies, observational studies and qualitative studies. |

| **Article** | **Addressed a clearly focussed issue** | **Appropriateness of method** | **Design** | **Recruitment** | **Relationship between researcher and participants** | **Ethical issues** | **Rigour of data analysis** | **Clarity of statement of findings** | **Overall** |
| --- | --- | --- | --- | --- | --- | --- | --- | --- | --- |
| Ancker et al 2009 | Low | Medium | Low | Medium | Low | Low | High | Medium | Medium |
| Bonner et al 2014 | Medium | Medium | Low | Medium | Medium | Low | Low | Low | Medium |
| Bonner et al 2014 | Low | Low | Medium | Low | Medium | Low | Low | Low | Low |
| Damman et al 2016 | Low | Low | Medium | Medium | Medium | Medium | Medium | Medium | Medium |
| Damman et al 2017 | Medium | High | High | Medium | Medium | Medium | Low | Medium | Medium |
| Goldman et al 2006 | Low | Low | Medium | Low | Medium | Medium | Low | High | Medium |
| Hill et al 2010 | Low | Low | Low | Medium | Medium | Low | Low | Low | Low |
| Middlemas et al 2014 | Low | Low | Medium | Medium | High | Medium | High | Medium | Medium |
| Shefer et al 2016 | Low | High | Medium | Low | Medium | Low | Low | Low | Medium |
| Sheridan et al 2009 | Medium | Medium | Medium | Medium | High | Medium | Medium | medium | medium |
| Wan et al 2008 | Medium | Medium | Medium | Medium | High | Medium | Medium | Medium | Medium |

**Table S2: Risk of bias for qualitative studies based on Critical Appraisal Skills Programme (CASP) appraisal tool**

| **Article** | **Addressed a clearly focused issue** | **Recruitment appropriate** | **Appropriate methodology** | **All enrolled participants accounted for** | **Groups similar and group assignment appropriate** | **treatment effect** | **Results applicable to local populations** | **All important outcomes considered** | **Results fit with other available evidence** |
| --- | --- | --- | --- | --- | --- | --- | --- | --- | --- |
| Adarkwah et al 2019 | Low | Low | Low | Low | Low | Low | Low | Medium | Medium |
| Bonner et al 2015 | Low | Low | Low | Low | Low | Low | High | Medium | Medium |
| Damman et al 2018 | Medium | High | High | Low | Medium | Medium | Medium | Medium | Low |
| Domenech et al 2016 | Medium | Medium | Medium | Low | Medium | High | Medium | Medium | Medium |
| Knowles et al 2017 | Low | Low | Low | Low | Low | Low | Medium | Medium | Medium |
| Lopez-Gonzalez et al 2015 | Low | Low | Low | Low | Medium | Low | Low | Low | Low |
| Naslund et al 2019 | High | High | High | High | Low | Medium | Medium | Medium | High |
| Powers et al 2011 | High | Medium | Medium | Medium | Low | Low | High | High | Medium |
| Ruiz et al 2013 | Medium | Medium | Medium | Medium | Low | Medium | High | Medium | Medium |
| Ruiz et al 2016 | Medium | Medium | Medium | Medium | Medium | Medium | High | Medium | Medium |
| Witteman et al 2014 | High | Medium | Low | Low | Low | Low | Medium | Medium | Low |
| Zikmund-Fisher et al 2014 | Low | Low | Medium | Low | Low | Medium | Low | Low | Medium |

**Table S3: Risk of bias for randomised and case controlled studies based on Critical Appraisal Skills Programme appraisal tools**

**Table S4: Risk of bias for all other quantitative studies based of Critical Appraisal Skills Programme appraisal tool**

| **Article** | **Addressed clearly focused issue** | **Recruitment** | **Exposure accurately measured to minimise bias** | **Outcome accurately measured to minimise bias** | **confounding factors** | **Follow up** | **How precise are the results** | **Do you believe the results** | **Can the results be applied to local populations** | **Results fit with the available evidence** | **Implications of study for practice** |
| --- | --- | --- | --- | --- | --- | --- | --- | --- | --- | --- | --- |
| Fair et al 2008 | Low | High | Medium | Medium | Low | Low | Low | Low | Medium | Medium | Medium |
| French et al 2004 | Medium | Medium | Medium | Low | High | Medium | Medium | Medium | Medium | Low | Medium |
| Frileux et al 2004 | Medium | Medium | Medium | Medium | Low | Low | Low | Medium | Medium | Medium | Medium |
| Johnson et al 2015 | Low | Low | Low | Medium | Medium | Medium | Medium | High | Low | Low | Low |
| Low Kalia et al 2006 | High | Low | Low | Medium | Medium | Low | Medium | Medium | Medium | Medium | Low |
| Korcarz et al 2008 | Medium | Low | Low | Medium | Medium | Low | Medium | High | Medium | Low | Low |
| Navar et al 2018 | Low | High | Medium | Medium | Medium | Medium | Medium | Medium | Medium | Medium | Medium |
| Orakzai et al 2008 | medium | medium | medium | High | High | Low | Medium | Medium | Medium | Low | Medium |

**Table S5 Factors during cardiovascular assessment which influence risk communication: participant quotes**

| Themes | | Participant Quotes |
| --- | --- | --- |
| Pre-assessment factors | Previous knowledge about cardiovascular disease | *“…disease that I’m not afraid of but if it would be about cancer or something like that, yes then I’d go to the doctor tomorrow, it’s just eh what frightens you, if it mentioned to you, what the diseases, yes what they can do to you and your body.”* (Damman et al., 2017)  *“because he’s called it CVD or cardiovascular disease, it’s too much for myself to take in but it seems if he said you’ve probably got a heart problem, that I haven’t got a problem with that because it’s easy to understand”* (Wan et al., 2008)  *“That test wouldn’t particularly motivate me because I already knew everything.”* (Damman et al., 2017) |
|  | Motivation to undergo cardiovascular risk assessment | *“I already knew that I’m not as risk an eh there’s nothing wrong with me, since you’d have to have complaints so really, yes for me this has no relevance”* (Damman et al., 2017) |
|  | Appropriateness of cardiovascular assessment | *“Cardiovascular risk just isn’t on their agenda, they’re more worried about mental health issues.”* (Bonner et al., 2014)  *“Too be honest if I talk too much they don’t turn up, they go to some other doctor.”* (Bonner et al., 2014) |
| Mode of Assessment | Doctor and patient relationships | *“I feel like doctors are intimidating… they kind of rush you.”* (Ancker et al., 2009)  *“You don’t want to seem stupid so you don’t ask.”* (Ancker et al., 2009) |
|  | Self-assessment | *“I think I’d be lower than that in reality”* participant who clicked default (Bonner et al., 2014) |
| Communication of results | Communication style | *“I like to put the fear into them… if they don’t pull up their socks bad things can happen to them”* (Bonner et al., 2014)  “Partnership not dictatorship” (Bonner et al., 2014)  *“Gives them a sense of empowerment, a bit of control” in relation to positive language* (Bonner et al., 2014) |
|  | Choosing strategy to communicate risk | *“You have to judge the people, at the time you have to make an informed decision as to how much information is going to sink in.”* (Bonner et al., 2014)  *“A lot of people I see don’t have great levels of literacy and that’s why the charts can be useful because they look at colours”* (Bonner et al., 2014) |
| Post assessment | Rationalising risk | *“because of stress”* (Middlemass et al., 2014)  *“Well I did the test and it turns out because of my family”* (Damman et al., 2017)  “*I reckon other people my age would probably be higher…two of my chums would definitely because they are overweight, they are on tablets”* (Bonner et al., 2014) |
|  | Reducing risk | “…taken more seriously by me than if I just dream it up on my own or its just handed to me.” Participant referring to agreeing a risk reduction plan with doctor. (Sheridan et al., 2009)  “this is quite good because it actually gives me targets for my BMI and what sort of weight I should be” reference to heart age (Bonner et al., 2014) |
|  | Reliability | “*I don’t know whether these tests could positively prove that that em if I have got a problem, it can’t be proved can it”* (Middlemass et al., 2014)  “I expected to fill in more about myself, about eating habits, smoking” (Damman et al., 2016)  *“just seems to simple that you can take a couple, four or five factors and come up with this percentage… I would say where did you get that information?”* (Sheridan et al., 2009)  *“How can you come up with a credible risk profile if factors like family history, exercise, stress not part of the calculation?”(Damman et al., 2017)* |
